# A Committee of Inquiry Appointed

**Published:** 1916-02-26

**Authors:** 


### NATIONAL INSURANCE ACT DEVELOPMENTS.

### A Committee of Inquiry Appointed.

An influential committee has at last been appointed by the Lords Commissioners of His Majesty's Treasury "to consider and report upon any amendments in the financial scheme of the National Insurance Acts which experience of the administration of sickness, disablement, and maternity benefits may suggest as desirable, within the existing limits of contributions and benefits and apart from further Exchequer grants, before the completion of any valuations of approved societies; and, further, to consider how far the work of approved societies could be simplified and its cost reduced, without detriment to the interests of insured persons, by amendment of the Acts and Regulations; and to make recommendations thereon."

An agitation for the appointment of such a committee has been on foot for some months past, the chief advocates in the House of Commons being Mr. Handel Booth and Mr. G. W. Currie. Until recently, however, the Government has turned a deaf ear to all requests for such inquiry, and only in December last we gathered from a speech of Mr. Charles Roberts, the Minister in charge of National Insurance matters, that it was contended that the time was inopportune for an inquiry. Since then various conferences of approved societies have adopted resolutions requesting the appointment of an inquiry, and the matter was brought to a head by a deputation, representing widely differing classes of approved societies, which waited on the Treasury on January 26, for on the very next day the announcement of the appointment was made, the terms of reference being as above stated.

### A Large Committee.

The committee is large in point of numbers, consisting of no fewer than twenty members, but by selecting so large a committee it has been possible to secure representation of all the most important interests involved. The Chairman, Sir Gerald Ryan, though not connected officially with National Insurance administration, took a keen interest in the formation of county associations of rural approved societies in the early days of the Act, and it is reputed that he has kept abreast of the developments that have since been made. He is not only a well-known actuary—having been president of the Institute of Actuaries—but is a business man with a reputation for "getting things done," and there is every reason to hope, therefore, that the labours of the committee, despite its size, will not be unduly prolonged.

It would be impossible in the space that is available to refer to each member of the committee in detail, and it would be invidious to make a selection. It is sufficient to say that the Insurance Commissioners are represented by six members, including the chief actuary to the Joint Committee of the Commissioners, the chairman and the president of the Institute of Actuaries represent the actuarial profession, while approved societies of various classes are represented by three members from trades unions, six members from friendly societies, two members from industrial assurance societies, and one member represents deposit societies. Among the members of the committee there are three ladies, who are already well known in connection with National Insurance matters. They will, presumably, take a special interest in amendments of the Acts relating to the insurance of women.

The only important class of approved societies which appears not to be specially represented is the class of small societies formed in connection with particular trades or by individual employers. The number of members in any one such society may not be large, but the number of societies of this description is considerable, so much so that the "federation" of the societies in question claims to represent half a million insured persons. If it is possible for the Treasury to give direct representation to this class of society upon the Committee of Inquiry, it would certainly seem desirable that it should do so, in order that the findings of the committee may be as comprehensive as possible, and to avoid the possibility of contentious matters being deferred until after the committee has reported. It may be hoping for too much to expect that the committee will be absolutely unanimous in their findings, but there would be no surer way of neutralising the good which it may be hoped the inquiry will effect than to defer until the report has been submitted to Parliament the discussion of matters affecting any considerable section of insured persons.

### The Terms of Reference.

With regard to the terms of reference, it will at once be seen that the primary object of the inquiry is to secure simplification. Simplification is necessary in the interests of economy, and the reduction of cost, without detriment to the interests of insured persons, is eminently desirable.

It is also clear from the terms of reference that the committee are not merely to consider matters of trifling detail, but they are required to take a comprehensive view of the administration of the Acts, being limited only to the present rates of contributions and benefits, and they are to recommend such amendments as they deem desirable, not only of the Regulations prescribed by the Commissioners, but also of the Acts themselves.

It will be noticed that the terms of reference do not include any mention of medical or sanatorium benefits, and the functions of Insurance Committees are not brought within the scope of the inquiry. To this extent the inquiry is not as comprehensive as it might have been, for there are admittedly many ways in which the administration of medical and sanatorium benefits might be improved; but there is ample work to be done in connection with the special functions of approved societies in the administration of the Acts, and it is as well, perhaps, that one well-defined section should be dealt with at a time. Moreover, it is apparent from the words employed in the terms of reference that the Treasury are desirous of obtaining a measure of reform before valuations have to be made. The valuations are in fact overdue, and there is evidently a desire in certain quarters to postpone them still further, in order that steps may be taken to avert the disclosure of deficiencies on any large scale.

It is not quite clear whether a fundamental change in the basis of finance—such, for instance, as the adoption of a plan under which it would not be necessary to accumulate reserves, or as affecting the relative proportions of contributions as between employer and employee—would be within the scope of the terms of reference. We venture to hope, however, that the widest possible interpretation will be made, so that the result of the inquiry may be absolutely the best in the interest of the vast numbers who are now embraced under the description of insured persons.

### Suggestions Invited.

Without doubt, the committee will hold formal sittings and hear witnesses in much the same manner as the Departmental Committee appointed to consider excessive sickness claims. The witnesses will be drawn from the officials of the various classes of approved societies, and they will speak from personal acquaintance with the details of the administration of the Acts. Their evidence will be very valuable, but it by no means follows that the evidence of careful observers who are not actually connected with the practical administration of approved societies would not be equally valuable. From some points of view it may even be that the engrossing detail will prevent those who are in constant daily touch with the matters concerned from taking a broad outlook in regard to amendments. In the hope, therefore, that we may be of some assistance to the inquiry, we shall be glad to receive suggestions from our readers on any practical matters connected with the administration of sickness, disablement, and maternity benefits. When the suggestions are judged to be of value we will have pleasure in giving public notice to them. We should welcome particularly suggestions from hospital governors and others with regard to the best method of securing adequate recognition of the valuable services rendered by the voluntary hospitals to insured persons. This is a matter which admittedly requires attention, and suggestions of a practical nature will be dealt with in a befitting manner, and, in view of the fact that the inquiry is of an open character, there seems to be ground for hope that really practical schemes may not only be courteously received, but that they may also be acted upon.

